# Depression, Hopelessness, and Complicated Grief in Survivors of Suicide

**DOI:** 10.3389/fpsyg.2018.00198

**Published:** 2018-03-08

**Authors:** Samantha Bellini, Denise Erbuto, Karl Andriessen, Mariantonietta Milelli, Marco Innamorati, David Lester, Gaia Sampogna, Andrea Fiorillo, Maurizio Pompili

**Affiliations:** ^1^Department of Neurosciences, Mental Health and Sensory Organs, Suicide Prevention Center, Sant’Andrea Hospital, Sapienza University of Rome, Rome, Italy; ^2^School of Psychiatry, University of New South Wales, Sydney, NSW, Australia; ^3^Department of Human Sciences, Università Europea di Roma, Rome, Italy; ^4^Stockton University, Galloway Township, NJ, United States; ^5^Department of Psychiatry, University of Campania “Luigi Vanvitelli” Naples, Naples, Italy

**Keywords:** complicated grief, depression, hopelessness, suicide, survivors

## Abstract

Suicide often has a severe impact on the surviving family and friends. There is a need to better understand the psychological and psychopathological consequences of losing a significant other by suicide. The aim of the present study was to assess hopelessness, depression, suicide risk, complicated grief, intrusive memories, and avoidance in a sample of suicide survivors. In this observational study, 35 bereaved individuals were recruited at the Suicide Prevention Centre of Sant’Andrea Hospital in Rome. Individuals were administered a series of validated instruments: the *Beck Depression Inventory II* (*BDI*), the *Beck Hopelessness Scale* (*BHS*), the *Inventory of Complicated Grief* (*ICG*), the *Impact of Event Scale* (*IES*), the *Subjective Happiness Scale* (*SHS*), and the *Satisfaction With Life Scale* (*SWLS*). Most survivors (62.8%) obtained high scores on measures of complicated grief. Scores on the measure of complicated grief were associated with intrusiveness of thoughts and memories, attempts to prevent the thoughts and emotions related to the event, depressive symptoms and hopelessness, and lower scores for feelings of happiness and satisfaction with life. A subgroup of suicide survivors may be at risk of severe psychological distress and suicidal behavior. Identification of these survivors is a necessary step for providing appropriate counseling and psychotherapy.

## Introduction

Suicide is a major public health problem, with more than 800,000 individuals dying by suicide annually (World Health Organization [WHO], 2014). It is estimated that for every suicide there are 6–10 people bereaved by the death (Cerel et al., 2008; Andriessen and Krysinska, 2012). In total, about 4.3% of the population has experienced a suicide in the past year, and 21.8% during their lifetime (Andriessen et al., 2017b). Hence, the population of suicide survivors, that is, the family members, friends, and others (e.g., colleagues, classmates, clinicians) who have lost someone by suicide, is the largest community of victims in the area of mental health related to suicide (Shneidman, 1969; Andriessen et al., 2017a).

Whereas grief is a normal, purposeful reaction to a loss, it is estimated that 7–10% of bereaved individuals may experience a condition known as “Complicated Grief” (Stroebe et al., 2013; Zisook et al., 2014; Shear, 2015). It is also referred to as Prolonged Grief Disorder (Prigerson et al., 2009), and more recently as Persistent Complex Bereavement Disorder (Robinaugh et al., 2014), a condition for further study. Although there is not yet a consensus about the exact set of diagnostic criteria and the name of the syndrome, it is typical of people who may experience major difficulties accepting the death of a significant other and its consequences. It is expressed through chronic, persisting characteristics of acute grief, and is more likely to occur after a sudden or violent death such as homicide or suicide (Lobb et al., 2010; Shear, 2015).

Some clinical characteristics of complicated grief may resemble symptoms of depression and post-traumatic stress disorder; however, there are a number of specific symptoms that allow reliable identification of complicated grief distinct from other disorders (Boelen, 2013; Stroebe et al., 2013; Zisook et al., 2014). Typical symptoms of complicated grief include intense yearning and longing for the deceased, intrusive thoughts or images about the deceased, rumination and intense feelings of anger and guilt (e.g., the feeling that they should have prevented the death), avoidance of situations, people and places that remind of the deceased, and difficulty finding meaning in life (Prigerson et al., 2009; Zisook et al., 2014; Shear, 2015). The bereaved individuals may feel numb and experience a diminished sense of self (Prigerson et al., 2009; Shear, 2015). Importantly, family and friends may become frustrated in their efforts to support the bereaved individual, which may increase their feelings of isolation and inadequacy (Shear, 2015).

Complicated grief may be associated with considerable morbidity, such as risk of cancer, cardiac events, sleep disturbances, and alcohol and substance abuse (Prigerson et al., 1996, 1997; Chen et al., 1999; Prigerson and Jacobs, 2001; Zisook et al., 2014). In addition, it is a risk factor for major depression, anxiety disorders, and suicidal ideation and behavior (Mitchell et al., 2005; Shear and Skritskaya, 2012; Zisook et al., 2014). Unlike the symptoms of reactive depression to bereavement, the symptoms of complicated grief can persist even after treatment with tricyclic antidepressants (Pasternak et al., 1991; Prigerson et al., 1996).

Longitudinal studies have shown that women are more at risk of depression, but not of complicated grief, after a loss by suicide than men (Saarinen et al., 2000; de Groot and Kollen, 2013). While levels of depression and grief symptoms can be elevated shortly after the loss, the risks of depression and complicated grief appear to decrease after the first year after the loss (de Groot and Kollen, 2013), and most symptoms have been found to disappear within 3 years after the loss, on average (Saarinen et al., 2000) or between 3 and 5 years (Feigelman et al., 2008–2009), indicating that risks of ill-mental health may subside after 3 years.

It is well-documented that those bereaved by suicide who experience high levels of functional and social impairments, and psychological distress, also experience barriers to help-seeking (Provini et al., 2000; Dyregrov, 2002). Depression/lack of energy, and lack of information about helpful resources have been identified as major barriers, whereas mental health professionals, amongst a variety of other helping professionals, have been experienced as most helpful by the bereaved (McMenamy et al., 2008). Given the potentially devastating effects of complicated grief on those bereaved by suicide it is crucial to assess their mental health.

### Aims

The study aims to assess the mental health of suicide survivors, i.e., their levels of complicated grief, comorbid depression and hopelessness, and suicidal ideation. The study will look at sex differences, time since loss (≤3 years vs. >3 years), and type of relationship (nuclear family vs. other relatives and friends). To the best of our knowledge, no such study has been conducted in our country; hence, the study was designed as a pilot study to test the feasibility of such assessment within a suicide prevention unit based in a hospital environment.

## Materials and Methods

### Sample

The Suicide Prevention Centre in Rome at Sant’Andrea University Hospital is a unique service in Italy, offering support for suicidal individuals as well as for those bereaved through suicide. Study participants were recruited between January and November 2014 through the bereavement support program, which includes participation in a psychological autopsy (Shneidman, 1981) to better understand a suicide death, and as a means of bereavement support. Participants were eligible if they were 18+ years old, and had lost at least one family member or close friend by suicide. Exclusion criteria were any psychiatric diagnosis in their medical history that could impair the evaluation of variables for the purpose of this study.

Thirty-five (17 males, 18 females) bereaved individuals participated in the study. The average age of participants was 47.3 years (range: 23–68; *SD* = 12.4). Two out of three participants (69%) were a first-degree family member of the deceased (partner, parent, son, sibling); the remaining were other relatives or friends. The majority (80%) had experienced one suicide loss, and the sociodemographic characteristics of the sample are reported in **Table 1**. Participation in the study was voluntarily, and each participant provided written informed consent; they were assessed in their living environment. Initially part of a dissertation project, the study protocol received ethics approval from the local research ethics review board.

**Table 1:** Sociodemographic characteristics of the sample (*n* = 35).

Variables	Frequencies (%)	Mean *(SD)*
Women	18 (51.4)	
Men	17 (48.6)	
Age	–	47.34 (12.39)
Years since the loss ≤ 3	13 (37.1)	
**Degree of kinship with the deceased**		
Partner	3 (8.6)	
Parent	9 (25.7)	
Son	9 (25.7)	
Sibling	3 (8.6)	
Other relative	9 (25.7)	
Friend	2 (5.7)	
**Number of suicides among relatives and friends**		
1	28 (80.0)	
>1	7 (20.0)	

### Measures

Clinical and sociodemographic characteristics of the sample were drawn from medical files independently by two researchers using a checklist, an checklist, and the data were compared for possible inconsistencies. All the participants were administered Italian versions of the *Beck Depression Inventory II* (*BDI-II*) (Beck et al., 1996; Ghisi et al., 2006), the *Beck Hopelessness Scale* (*BHS*) (Beck et al., 1974; Pompili et al., 2007, 2009; Innamorati et al., 2013), the *Inventory of Complicated Grief* (*ICG*) (Prigerson et al., 1995; Carmassi et al., 2014), the *Impact of Event Scale* (*IES*) (Horowitz et al., 1979; Pietrantonio et al., 2003), the *Subjective Happiness Scale* (*SHS*) (Lyubomirsky and Lepper, 1999; Iani et al., 2014), and the *Satisfaction With Life Scale* (*SWLS*) (Diener et al., 1985; Pavot et al., 1991; Di Fabio and Gori, 2015). Cronbach alphas for the present sample are reported in **Table 3**.

The BDI-II (Beck et al., 1996) assesses the presence and severity of depressive symptoms according to DSM-IV criteria. A total score of 0–13 is considered as the absence of or minimal depression, 14–19 mild depression, 20–28 and 29–63, respectively, moderate and severe depression. Good estimates of internal consistency and concurrent validity have been demonstrated for the Italian version of the BDI-II (Ghisi et al., 2006).

The BHS (Beck et al., 1974) evaluates the presence of despair and negative expectations about the future (examples of items, “I look forward to the future with hope and enthusiasm” [reverse scored], “My future seems dark to me”) and indicates the risk of suicide (Beck et al., 1985; Beck and Steer, 1988). Scores range from a minimum of 0 to a maximum of 20, with higher scores indicating more severe hopelessness. A cut-off score of 9 indicates the presence of severe hopelessness.

The ICG (Prigerson et al., 1995) assesses indicators of pathological grief (examples of items, “I feel lonely a great deal of the time ever since he/she died,” “I hear the voice of the person who died speak to me”), such as anger, disbelief, and hallucinations. Higher scores indicate the presence of more severe pathological grief, and scores of 25 and higher indicate important difficulties in daily life associated with the memories of the event.

The IES (Horowitz et al., 1979) measures current subjective distress related to a specific event using the dimensions of intrusion and avoidance (example of items, “Any reminders brought back feelings about it”).

The SHS (Lyubomirsky and Lepper, 1999) is a four-item scale measuring subjective happiness (example of items, “Some people are generally very happy. They enjoy life regardless of what is going on, getting the most out of everything. To what extent does this characterization describe you?”) with higher scores indicating greater happiness.

The SWLS (Diener et al., 1985; Pavot et al., 1991) is a five-item instrument assessing satisfaction with one’s own life (example of items, “In most ways my life is close to my ideal”). Higher scores indicate greater satisfaction with life.

### Analysis

All the analyses were performed with SPSS for Windows version 17. Correlations between variables were reported as Spearman *rho* correlations coefficients. Partial correlations between pairs of variables were computed while controlling for sociodemographic variables (age, sex, time since loss, and number of losses from suicide). Mann–Whitney *U*-tests were used for comparisons between groups (>3 years vs. <3 years from the loss, and 1 loss vs. >1 losses from suicide).

## Results

Mean time since loss was 3.20 years (*SD* = 1.41; median = 3; interquartile range = 3). **Table 2** presents the results from the psychological tests. More than 51% of the sample reported severe hopelessness, 28.6% moderate to severe depression, and 62.8% indicated difficulties in daily life associated with the memories of the event. Neither time since loss nor number of losses from suicide were significantly associated with psychological variables (all Mann–Whitney tests were non-significant; *p* > 0.05). Nevertheless, we calculated partial correlation coefficients among psychological variables also controlling for these variables. The presence and severity of complicated grief was associated with more intrusive thoughts and memories (ρ = 0.60; *p* < 0.01) and avoidance of thoughts and emotions related to the event (ρ = 0.42; *p* < 0.05), more severe depression (ρ = 0.53; *p* < 0.01) and hopelessness (ρ = 0.54; *p* < 0.01), and less subjective happiness (ρ = -0.60; *p* < 0.01) and satisfaction with life (ρ = -0.57; *p* < 0.01) (see **Table 3**). Partial correlations indicated that age and sex did not mediate the relationships of complicated grief with other psychological variables, while the inclusion in the analyses of the number of losses from suicide was able to mediate partially these relationships. The inclusion of number of losses from suicide attenuated the relationships of complicated grief with intrusion (ρ from 0.60 to 0.54; *p* < 0.01) and avoidance (ρ from 0.42 to 0.34; *p* < 0.05) of thoughts and emotions related to the event, and amplified the inverse relationship between complicated grief and subjective happiness (ρ from -0.60 to -0.67; *p* < 0.01). Small changes in the magnitude of the relationships when the number of losses from suicide was included in the analyses were evident also for the association between complicated grief and hopelessness and depression.

**Table 2:** Psychological characteristics of the sample (*n* = 35).

Variables	Percentages	Mean *(SD)*
IES Intrusion	–	10.14 (4.69)
IES Avoidance	–	8.09 (4.38)
ICG	–	28.29 (15.37)
ICG > 25	62.8	–
BHS	–	9.00 (5.25)
BHS ≥ 9	51.4	–
BDI	–	14.71 (10.37)
BDI ≥ 20	28.6	–
SHS	–	15.37 (5.11)
SWLS	–	18.89 (7.96)

*IES, Impact of Event Scale; ICG, Inventory of Complicated Grief; BHS, Beck Hopelessness Scale; BDI, Beck Depression Inventory II; SHS, Subjective Happiness Scale; SWLS, Satisfaction With Life Scale*.

**Table 3:** Correlations between complicated grief and other variables (*n* = 35).

	ICG	Intrusion	Avoidance	BHS	BDI	SHS	SWLS
ICG	–	0.60^∗∗^ (0.60^1^;0.60^2^; 0.58^3^;0.54^4^)	0.42^∗^ (0.42^1^;0.42^2^; 0.40^3^;0.34^4^)	0.54^∗∗^ (0.54^1^;0.54^2^; 0.53^3^;0.57^4^)	0.53^∗∗^ (0.53^1^;0.52^2^; 0.50^3^;0.54^4^)	-0.60^∗∗^ (-0.60^1^;-0.60^2^; -0.59^3^;-0.67^4^)	-0.57^∗∗^ (-0.57^1^;-0.57^2^; -0.57^3^;-0.60^4^)
Cronbach alpha	0.93			0.88	0.91	0.81	0.92

^∗∗^*Correlation is significant at the 0.01 level (two-tailed); ^∗^correlation is significant at the 0.05 level (two-tailed). Partial correlations controlling for: ^*1*^age; ^*2*^age and sex; ^*3*^age, sex, and time since the loss; ^*4*^age, sex, time since the loss, and number of losses through suicide. IES, Impact of Event Scale; ICG, Inventory of Complicated Grief; BHS, Beck Hopelessness Scale; BDI, Beck Depression Inventory II; SHS, Subjective Happiness Scale; SWLS, Satisfaction With Life Scale*.

## Discussion

The present study explored the mental health of bereaved individuals who had lost a significant other to suicide. The study revealed that 63% of those bereaved had elevated scores on a measure of complicated grief, and that complicated grief scores correlated moderately but positively with depression and hopelessness and negatively with subjective happiness and satisfaction with life. Most of the participants experienced high levels of depression and hopelessness, fewer positive expectations for the future, major symptoms of complicated grief, accompanied by lower perceived life satisfaction.

These findings shed light on a vulnerable population far greater than the number of suicides, i.e., those who experience a painful grief process. The cross-sectional nature of the study does not permit conclusions about the causes of the distress, yet it is apparent that distress can be present even many years after the death. Recently, scholars (Feigelman et al., 2018) reported how pervasive suicide exposure and bereavement is, pointing to a far greater percentage of individuals being touched by such phenomenon. These authors pointed out that suicide bereavement may affect up to more than a third of the US population.

These survivors may be at increased risk of suicidal behavior, and their identification by means of validated instruments is a first crucial step in order to facilitate appropriate psychotherapeutic support (Shear et al., 2005; Wittouck et al., 2011).

The high levels of symptoms of complicated grief, even several years after the death, as well as the significant association between the level of complicated grief and the level of intrusiveness and avoidance on the IES scale, which are characteristic of PTSD, suggest that, in the case of suicide, it is hard for the bereaved to achieve a level of acceptance and emotional balance (Maciejewski et al., 2007). In addition, the entire sample presented high levels of hopelessness is only pointing to reduced future expectations. Beck et al. (1996) reported that hopelessness is a better predictor of fatal and non-fatal suicidal behavior than depression. Although, hopelessness is only a proxy of suicide risk, we found that more than half of the participants had such risk. This finding confirms research results which have found an increased risk of suicidal behavior among family members who have experienced the suicide of a significant other (Pitman et al., 2014, 2016). It is important, therefore, to provide psychiatric screening for those bereaved by suicide as well as to support caregivers who deal with suicidal individuals (McLaughlin et al., 2016). If survivors were to participate in treatment, they could expect to experience benefits, such as lower levels of complicated grief, depression, and a higher perceived quality of life (Shear et al., 2005; Wittouck et al., 2011).

Research into the effectiveness of suicide bereavement support suggests that there is no ‘one size fits all.’ Those bereaved who are in need of sharing their experiences and finding social support appear to benefit from participation in support groups, whereas those who struggle with psychiatric or psychological issues might be better served through psychotherapy (McIntosh, 2017). Systematic reviews of the literature have found modest, yet positive evidence of effectiveness of group and family interventions regarding dealing with grief, depression and other mental distress (McDaid et al., 2008; Szumilas and Kutcher, 2011).

Obviously, the provision of therapeutic support in an individual case will depend on the scholarly background of the therapist, the needs of the bereaved, their social support and family context (Andriessen and Krysinska, 2016). It is, however, advised that therapy for individuals or families would be provided in a supportive and educational climate. An involved, humanistic, compassionate approach would be preferred to overly directive or passive approaches. Assessment, addressing grief reactions and the creation of a ‘suicide story’ have been identified as crucial therapeutic aspects (Andriessen and Krysinska, 2016). Working with those bereaved by suicide may be emotionally demanding for the psychotherapist. Hence, professional education, peer counseling, or supervision for those therapists may be indicated (Castelli Dransart et al., 2017).

The present study did have some limitations, including its cross-sectional nature, a small sample size, and lack of comparison group which may over-represent the impact of survivorship and related symptoms. However, we observed that those bereaved by suicide are often ambivalent about their willingness to share their experiences with health professionals. Those bereaved may be a silent and hidden population. Hence, even the exploration of small samples can contribute to a better understanding of their mental health. Another limitation relates to the fact that we did not have access to information regarding the pre-suicide psychological status of the bereaved nor was a psychiatric assessment performed through clinical interview or structured diagnostic interview. Furthermore, some individuals had looked for mental health assistance, and this may have biased the percentages and the reporting of symptoms. We also acknowledge the lack of a control group which would have provided more solid results for this observational study.

Despite these limitations, the results point to the need to further study the phenomenology of complicated grief in suicide survivors. Further studies may include larger samples and control groups (bereaved by non-suicide deaths as well as non-bereaved controls). Also, measurements before and after participation in the support program may further improve our insights in the needs of the bereaved and the support offered. The current study has shown that it is possible to assess the mental health, including depression and complicated grief, of people bereaved by suicide at a Suicide Prevention Centre. Such an assessment may facilitate help-seeking among those bereaved who are vulnerable for adverse mental health outcomes and suicidality.

## Author Contributions

MP provided the design of the study. SB, DE, and MM collected the data and provided first draft. AF, DL, GS, and KA contributed to the drafts and interpretation of the results. MI provided the statistical analysis. All authors contributed in drafting the final version of the paper.

## Conflict of Interest Statement

The authors declare that the research was conducted in the absence of any commercial or financial relationships that could be construed as a potential conflict of interest.
